# Novel 3-Dimensional K-Space segmented acquisition scheme (CENTRA-PLUS) for enhanced coronary imaging

**DOI:** 10.1186/1532-429X-18-S1-P112

**Published:** 2016-01-27

**Authors:** Mita Patel, Amit R Patel, Hui Wang, Donovan Gorre, Roberto Lang, Keigo Kawaji

**Affiliations:** 1 grid.170205.10000000419367822Cardiology, University of Chicago, Chicago, IL USA; 2 Phillips Healthcare, Cleveland, OH USA

## Background

**Figure 1:**
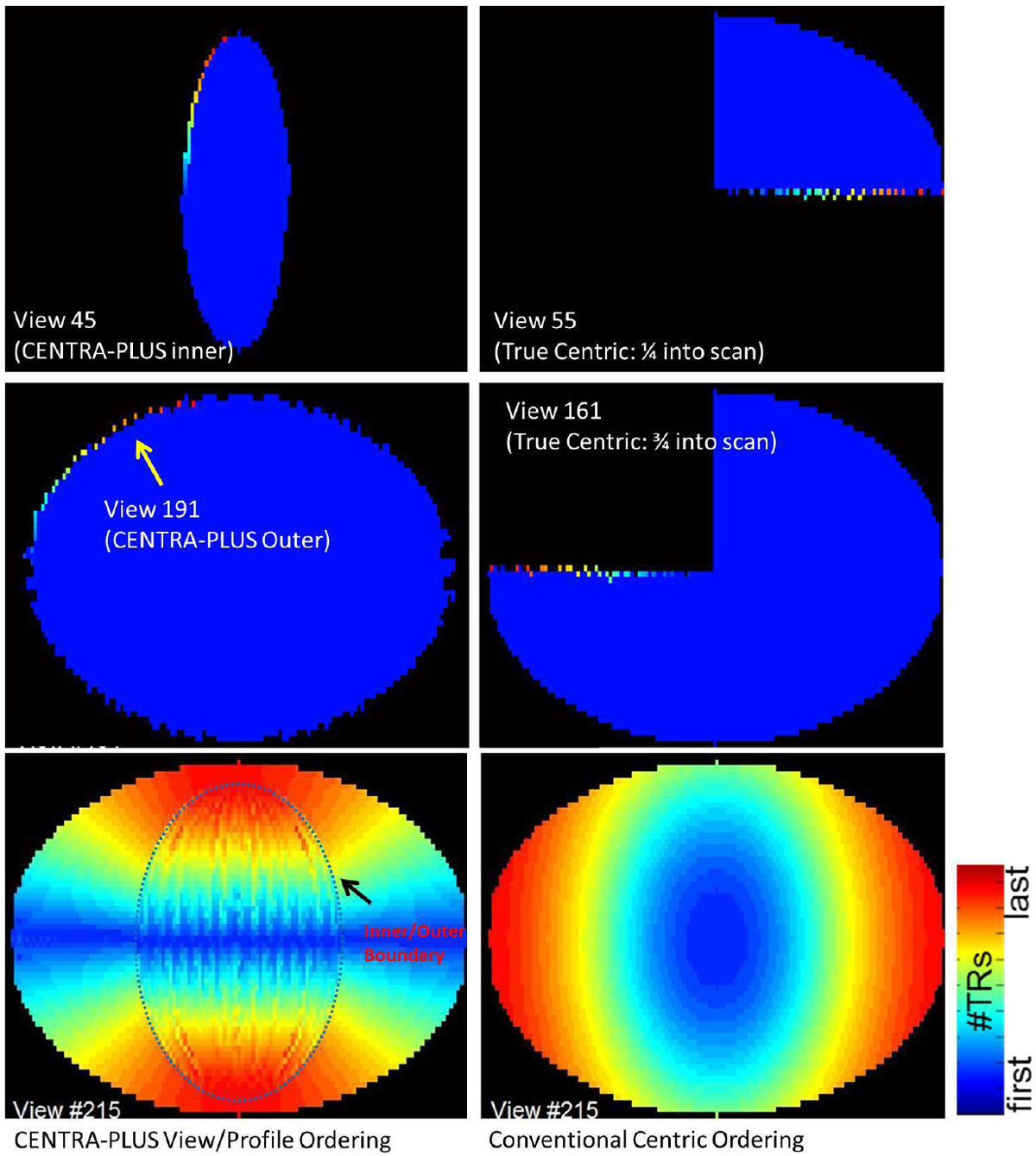
**Comparison between CENTRA-PLUS and Conventional Centric k-space View/Profile Ordering**. CENTRA-PLUS allows for flexible outer k-space navigator gating.

Bright-blood coronary artery imaging by cardiovascular magnetic resonance remains a challenge due to several factors including coronary tortuosity, cardiac and respiratory motion, and high demands in spatial resolution and image contrast. Cartesian CENTRA-PLUS for high-resolution 3D k-space acquisition sorts the different regions of segmented k-space at different time-points of the ~5 minute scan (figure 1). This strategy is well-suited for various acceleration methods including GRAPPA, compressed sensing, and navigator strategies for scan time reduction that conventional centric acquisition strategies do not easily allow. The aim of this study is to perform a direct comparison of CENTRA-PLUS against the conventional centric acquisition for coronary CMR.

## Methods

We studied ten healthy volunteers with both CENTRA-PLUS and conventional centric acquisition strategies matched for all other acquisition parameters. Scans were performed on a 1.5T Philips Achieva with a 5ch array (TR = 4.4 ms; TE = 1.9 ms; FA = 90; 300 × 300 × 120 mm^3^ acquired at 1.3-1.5 mm^3^ isotropic resolution, interpolated to 0.65 × 0.65 × 1.3 mm, sensitive encoding parallel imaging (r=2)). A fixed 5 mm gating window with no slice tracking was used. 5-point qualitative scores of coronary artery visualization (1= not visible, 5= excellent) and quantitative parameters including Signal-to-Noise-ratio (SNR) and Contrast-to-Noise-ratio (CNR) were evaluated using a fast noise scan for SENSE-accelerated analysis. Statistical analysis of SNR, CNR, and scan time was performed using Student's t-test and qualitative scores were assessed using Wilcoxon's Signed-Rank test.

## Results

**Figure 2:**
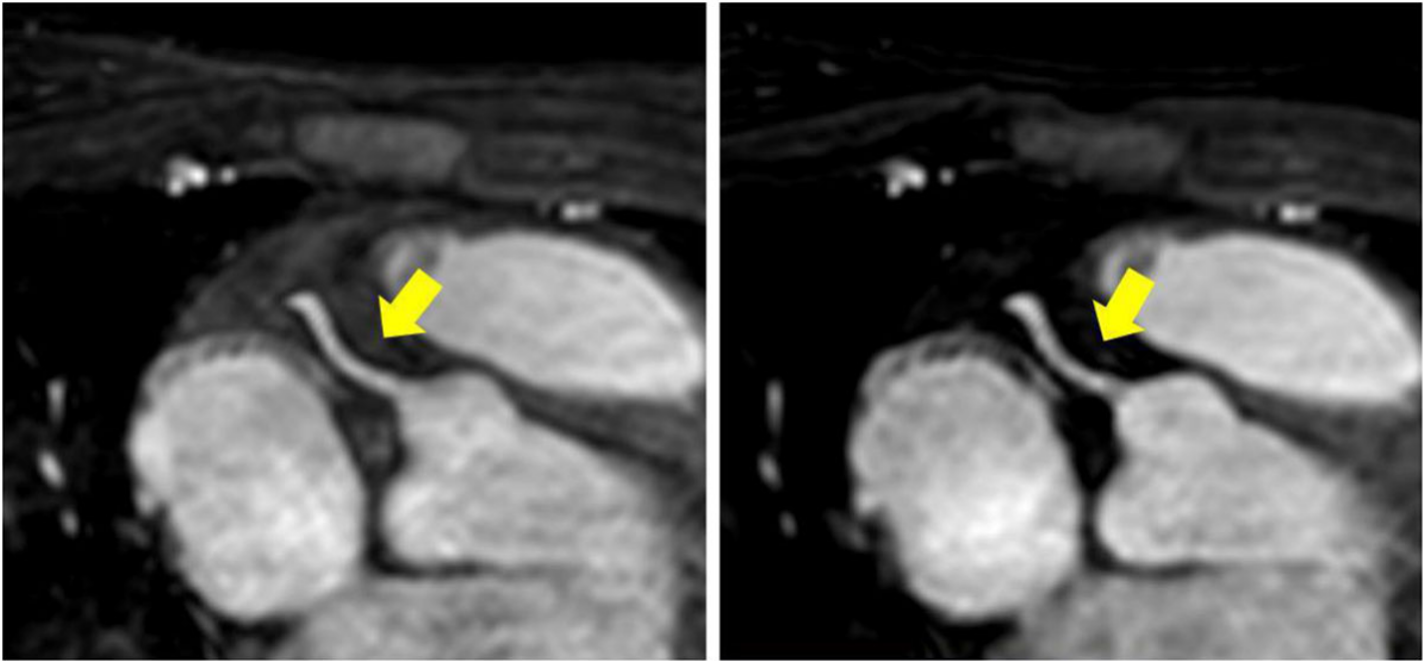
**RCA visualization from one subject using: Left) CENTRA-PLUS and Right) Conventional Centric View/Profile Ordering**.

Qualitative scores for vessel visualization using CENTRA-PLUS were improved compared to the conventional centric acquisition strategy (3.0 ± 0.8 vs 2.7 ± 0.8, p< 0.05). In addition, there was an improvement in SNR of the right coronary artery (RCA) using CENTRA-PLUS (98 ± 16 vs 88 ± 16, p < 0.002) (figure 2). There was no significant difference in SNR of the left anterior descending (LAD) and left circumflex artery (LCX). The CNR of the RCA, LAD, and LCX were also comparable between both strategies (p = ns). Furthermore, there was no significant difference in scan time between the centric coronary MRA acquisition and CENTRA-PLUS (518 ± 187s vs 437 ± 90s, p = ns) with matched navigator gating strategy.

## Conclusions

CENTRA-PLUS is a promising acquisition scheme for coronary CMR resulting in improved image quality and SNR.

